# Nasopharyngeal Bacterial Carriage in the Conjugate Vaccine Era with a Focus on Pneumococci

**DOI:** 10.1155/2015/394368

**Published:** 2015-08-16

**Authors:** V. T. Devine, J. M. Jefferies, S. C. Clarke, S. N. Faust

**Affiliations:** ^1^Academic Unit of Clinical and Experimental Sciences, Faculty of Medicine and Institute for Life Sciences, University of Southampton, Southampton SO16 6YD, UK; ^2^University of Southampton Malaysia Campus, 79200 Nusajaya, Malaysia; ^3^Southampton NIHR Wellcome Trust Clinical Research Facility and Respiratory Biomedical Research Unit, University Hospital Southampton NHS Foundation Trust, Southampton SO16 6YD, UK

## Abstract

Seven-valent pneumococcal conjugate vaccine (PCV7) was included in the UK national immunisation program in 2006, and this was replaced by thirteen-valent PCV in 2010. During this time, the carriage of vaccine-type *Streptococcus pneumoniae* decreased but pneumococcal carriage remained stable due to increases in non-vaccine-type *S. pneumoniae*. Carriage studies have been undertaken in various countries to monitor vaccine-type replacement and to help predict the serotypes, which may cause invasive disease. There has been less focus on how conjugate vaccines indirectly affect colonization of other nasopharyngeal bacteria. If the nasopharynx is treated as a niche, then bacterial dynamics are accepted to occur. Alterations in these dynamics have been shown due to seasonal changes, antibiotic use, and sibling/day care interaction. It has been shown that, following PCV7 introduction, an eradication of pneumococcal vaccine types has resulted in increases in the abundance of other respiratory pathogens including *Haemophilus influenzae* and *Staphylococcus aureus*. These changes are difficult to attribute to PCV7 introduction alone and these studies do not account for further changes due to PCV13 implementation. This review aims to describe nasopharyngeal cocarriage of respiratory pathogens in the PCV era.

## 1. Introduction

Invasive pneumococcal disease (IPD) is a cause of substantial morbidity and mortality worldwide, with over 5,000 cases reported in the UK per year [1]. For patients with IPD in the United States, around 10% will die from the illness [2]. In 2008, globally there were an estimated 476,000 deaths attributed to pneumococcal infection among children less than five years of age. As of 2014, globally 59% of infants still live in countries where a PCV has yet to be added to the national immunisation program [3]. The seven-valent pneumococcal conjugate vaccine (PCV7) (Prevenar, Pfizer, previously Wyeth) was added to the US immunisation schedule in 2000 and to the UK immunisation schedule in 2006. The effect of PCV7 on pneumococcal carriage in children has been investigated, with vaccine serotypes decreasing since PCV introduction [4–6]. In the UK and elsewhere, the incidence of IPD has decreased after the implementation of PCVs [7–10]. As PCV13 was introduced, the rates of vaccine-type carriage and IPD have similarly declined [11–13], and as additional higher valency PCVs are introduced it is expected that IPD incidence will continue to decrease [14].

The effect of pneumococcal vaccination on other bacterial species known to occupy the same niche as* S. pneumoniae* has not been fully investigated, in particular determination of how changes in the human microbiome can be attributed to external pressures or vaccine introductions [15, 16]. This paper reviews what is known about cocarriage of nasopharyngeal bacteria.

## 2. Pneumococcal Disease

*S. pneumoniae* are Gram-positive diplococci often found to occupy the nasopharynx. Pneumococci are typed according to the serological response to their external polysaccharide capsule. Strains of* S. pneumoniae* that do not react with type-specific antisera are deemed nontypeable (NT)* S. pneumoniae*. Currently 94 pneumococcal serotypes have been characterised [17–22]. Individuals become colonised with* S. pneumoniae* and other nasopharyngeal flora during their first few months of life [23], although the age of pneumococcal colonisation varies and may be attributed to environmental factors such as having siblings, attending day care, or geographical location [23, 24]. Colonisation with a pneumococcal isolate is a prerequisite for pneumococcal infection; the capsule type of* S. pneumoniae* rather than genotype is thought to modulate the degree of infection [25]. The capsular type of the pneumococcus dictates the duration of colonization in children with common serotypes retained longer in carriage. As the age of a child increases, so does their immune mediated clearance of pneumococcal serotypes [26]. Disease caused by pneumococcal infections can be divided into two groups: invasive pneumococcal disease (IPD) and noninvasive disease.

## 3. Immunisation to Reduce Disease Burden

The 23-valent pneumococcal polysaccharide vaccine (PPV23) (Pneumovax II, Aventis Pasteur) is a plain polysaccharide vaccine that induces an immune response to the polysaccharide capsule of an infectious organism to induce short-term memory B-cells and antibody production. As the immune response produced is classed as “slow, no immune memory,” this type of vaccine is not effective in young children and infants (IPD risk groups). T-cell independent vaccines also appear not to prevent carriage of the bacterial species [27] after the short-lived immune response has finished.

Conjugate vaccines contain bacterial polysaccharides from the outer capsule of an organism, for example, PCV7 (seven-valent pneumococcal conjugate vaccine, Prevenar, Pfizer); 10-valent PCV (Synflorix, GSK) and 13-valent PCV (Prevenar 13, Pfizer) are currently licensed pneumococcal conjugate vaccines. The polysaccharide is converted into a T-cell dependent antigen through the presence of the carrier protein. Long-term memory B-cells mature so that the immune system has both a short-term and long-term response invoked when those polysaccharides are encountered again. This reduces colonisation of the serotypes included within the vaccine, helping to prevent infection even in the very young. PCVs are given to children rather than a PPV to produce a stronger and more long-lasting immune response [28] and PCVs have also been found to be more effective against vaccine serotypes than PPV in older adults [29]. The 10-valent pneumococcal vaccine, PHiD-CV10 (GSK), also includes conjugation of nontypeable* Haemophilus influenzae* protein D. PCV10 is comparable to PCV7 at preventing invasive pneumococcal disease [30] and has been found to have a higher immunologic coverage for acute otitis media than PCV13 [31].

## 4. Pneumococcal Conjugate Vaccination

Prior to implementation of 7-valent pneumococcal conjugate vaccination, the majority of invasive disease globally was caused by seven of the pneumococcal serotypes [32]. PCV13 introduction has further addressed IPD caused by the 6 serotypes included in the new vaccine in Europe and North America [33]. PCV effectiveness is subject to strains undergoing capsular changes including (a) serotype replacement/shifting [34], where prevalence of a nonvaccine serotype increases as prevalence of a vaccine serotype decreases and the non-vaccine-type bacteria overcome vaccine challenges in a community [35], and (b) capsular switching, where an individual bacterium can undergo changes in the capsular genes, causing the bacteria to change serotype [36]. Through alteration of capsular expression and the increase in prevalence of serotypes not included in vaccine formulations, serotypes in carriage may be replaced with more virulent serotypes [37]. However it has been reported that capsular switching resulting from vaccine pressures will only contribute to an increase of a maximum of three extra cases of IPD per 100,000 vaccinated children cumulated over a ten-year period [38]. The additional maximum of three cases per year was deduced using a mathematical model of pneumococcal transmission based on IPD data presented in previous European publications [38]. However until there is evidence from more studies of capsular switching after PCV, IPD from non-vaccine-type pneumococci may be a more pressing issue [39]. Antibiotic resistance in pneumococcal isolates has also been shown to be present globally in both carriage [40–42] and disease [43–45] cases. Reasons for pneumococcal antibiotic resistance include vaccine pressures as well as overprescribing and overuse of antibiotics acting as a selective pressure for current strains to undergo clonal expansion [46, 47].

## 5. Interactions of Nasopharyngeal Microbiota

The microbiota of the human nasopharynx contains both commensal and potentially pathogenic species with external environmental factors and the presence of antibiotic resistant species contributing to disease states [48].

Bacteria found to reside in the nasopharynx other than* S. pneumoniae* include* H. influenzae*,* Moraxella catarrhalis*, alpha-haemolytic streptococci (*α*-HS),* Staphylococcus aureus*, and* Neisseria meningitidis* which are included in this review as respiratory bacteria capable of causing significant infections.* M. catarrhalis* is a nonmotile Gram-negative human commensal and opportunistic pathogen responsible for a range of infections, including causing an estimated 10% of adult chronic obstructive airways disease (COPD) exacerbations [49]. It has been shown that even the colonisation of* M. catarrhalis* in a COPD patient can contribute to the progression of airway disease [50].* S. pneumoniae*,* Streptococcus mutans*, and* Streptococcus sanguis* are all species of *α*-haemolytic streptococci (*α*-HS) that are both commensal and pathogenic in people;* Streptococcus viridans* are a group of streptococcal organisms including* Streptococcus mitis*,* Streptococcus salivarius*, and* S. mutans*.* S. viridians* have been shown to be involved in causing empyema thoracis and lung abscesses [51].* S. aureus* is a commensally carried Gram-positive bacterium that can act as an opportunistic respiratory pathogen in susceptible individuals. Nasal carriage of* S. aureus* is positively associated with noninvasive and invasive infections, compared to those who do not carry* S. aureus* [52]. It is important to assess the carriage of* S. aureus* and to monitor if carriage of methicillin-resistant (MRSA) bacteria is increasing in the community. Carriage of the Gram-negative diplococcus* N. meningitidis* is higher in young children under 1 year of age and then, after 15 years of age [53],* N. meningitidis* can result in a wide range of infections and bacterial load is associated with mortality, particularly for serogroup C that produces a higher bacterial load in patients [54].* H. influenzae* are Gram-negative coccobacilli, serologically typed a–f, as well as a large, distinct population [55] that are unencapsulated, termed nontypeable* H. influenzae* (NTHi). Certain bacterial genes of NTHi have been found to be associated to aid bacterial persistence within the lower airways of patients with COPD [56] as well as now being known to cause invasive disease in risk groups [57].

## 6. Monitoring Bacterial Carriage

Bacterial colonization is thought to be a prerequisite for an individual to become infected, but bacterial colonisation does not normally result in infection [58]. There are many relevant studies, both completed and ongoing, that monitor bacterial carriage in individuals [59–63]. Bacterial carriage may be monitored to detect before and after changes following the implementation of a preventative vaccination strategy [4, 59, 64–68]. Carriage can be monitored for changes attributed to age, health status, geographical location, ethnicity, and many other environmental factors [69, 70]. Carriage of a number of bacteria or carriage of a single bacterial species can be monitored. Respiratory bacteria can be detected using relatively noninvasive means such as a nasopharyngeal [71, 72] or nose swab, meaning that larger numbers of patients can be recruited to strengthen the results gained from the study.

Pneumococcal carriage studies are highly informative and beneficial for a number of reasons. Through surveillance of invasive disease studies, we have seen how PCVs are effective against invasive vaccine-type (VT) pneumococcal disease [73], and through carriage studies we can see the reduction of VT pneumococcal colonisation and VT pneumococcal transmission [74]. It is also possible to monitor indirect effects of PCVs (Table 1), such as changes in dynamics of the nasopharynx to detect microbial shifts [75], where the bacterial species change in content or numbers due to an external pressure.

## 7. A Niche of Carriage and Infection

Bacterial cocolonisation of the nasopharynx niche can be classed as dynamic as it comprises both synergistic and competitive associations. These associations can change depending on whether or not the niche is in a healthy or a disease state [76]. A lower diversity of nasopharyngeal flora has been positively associated with higher carriage rates of nasopharyngeal pathogens including* S. pneumoniae*,* H. influenzae*, and* M. catarrhalis* [77]. Individuals with spontaneous otorrhea that have multiple pneumococcal serotypes colonising at any one time were more likely to present with other species cocolonising [78]. Viral infection has been to leave the middle ear vulnerable to infection by bacteria that normally reside in the nasopharynx [79].

External factors such as sibling interaction and interaction with other children can play a part in polymicrobial carriage, and such relationships are associated with more frequent nasopharyngeal carriage of potential pathogens [80]. Adult associations with more frequent carriage of potential pathogenic species include, but are not limited to, the presence of children either at home or at work, preexisting allergic conditions, and respiratory conditions including COPD and asthma [80]. Such data imply that children are reservoirs for bacterial pathogens. With increased contact between children and other children or children and adults, there is a greater chance for bacterial transmission between the individuals.

*S. pneumoniae* and* H. influenzae* are frequently found to cocolonise the nasopharynx, and competition may exist between the two organisms for nutritional resources and for dominance of the niche. It has been shown that* H. influenzae* when colonising with* S. pneumoniae* may outcompete them for survival through signaling of nucleotide-binding oligomerisation domain-1 (Nod1) to facilitate clearance of* S. pneumoniae* [81], but virulent* S. pneumoniae* serotypes show resistance to host cell-mediated clearance as a mechanism to overcome these attacks [82]. Both organisms cause immune responses in colonised individuals and cocolonisation by these two pathogens can result in exaggerated immune responses with prolonged hospitalization particularly for young asthmatics experiencing their first count of wheezes [83].

Before the inclusion of the* H. influenzae* type b (Hib) conjugate vaccine in the UK routine paediatric immunisation schedule in 1992 [84], around 95% of invasive* H. influenzae* disease was attributed to serotype b alone [85]. After Hib vaccination there was a dramatic 98% decrease in invasive disease by 1998 [86]. However a small amount of Hib disease has still been reported in some vaccinated populations, ranging from invasive disease due to vaccine failure in the UK and elsewhere [86, 87]. Increased disease incidence has been reported for non-Hib serotypes [88–90]. NTHi has also been reported as a cause for invasive disease [90, 91]. The surveillance following Hib vaccination indicates that vaccine-type replacement is seen with other strains not included in vaccine formulation; however studies have not yet fully elucidated the effects on cocolonising niche species.

Polymicrobial cocarriage can consist of more than one bacterial species; this can also include viruses and fungi [92]. A polymicrobial infection combining both bacteria and fungi can mount a greater immune response within a host than infection by either bacteria or fungi [93]. Preinfection with a virus can destroy epithelial cells and allow better adhesion for bacteria, thus priming the middle ear for further infection that can result in otitis media [94]. Bacterial communities that aggregate together on a surface are known as biofilms. Biofilms have a number of mechanisms to increase persistence and survive, which protect from both therapeutic attack and host immune responses [95, 96]. Bacterial biofilms contribute to a range of chronic respiratory and otolaryngeal diseases. Bacterial biofilms are commonly detected and implicated in pathogenicity in children with recurrent acute otitis media [97, 98], chronic middle ear effusion [99], and other chronic respiratory conditions.

## 8. Indirect Effects of PCV Implementation

Reports are emerging of the effect of PCV implementation on cocarriage and disease caused by bacteria other than pneumococci. Where vaccine serotypes of* S. pneumoniae* have been eradicated, there has been an increase in nontypeable* H. influenzae* isolated in cases of otitis media [100]. A large study of healthy children and children with recurrent otitis media, all less than 36 months of age in Western Australia, has shown that with a decrease in* S. pneumoniae* and* S. pneumoniae* PCV7 VT serotypes there is a corresponding increase in* H. influenzae,* particularly NTHi. Another study has shown that colonisation with of* S. pneumoniae *invasive serotype 19A is associated with a decrease in colonisation of* H. influenzae* [101]. To study the indirect effects of PCV implementation without introducing bias through standard microbiology culture, 16S-sequencing was used to sequence nasopharyngeal swabs of children with or without otitis media, which demonstrated that an infection is associated with increased* S. pneumoniae* and* H. influenzae* being present with a lack of protective flora present [102]. Another study set out to characterize the nasopharyngeal niche to deduce which, if any, external factors (such as viral carriage and day care level) have an effect on the microbiota of the nasopharynx of young children. Results showed that seasonal changes were occurring but that these were unrelated to viral or antibiotic causes and that seasonal variations corresponded to “healthy” probiotic species being more abundant in summer rather than autumn [103].

## 9. Summary

Pneumococcal conjugate vaccines (PCVs) affect the carriage of* S. pneumoniae* and the carriage of vaccine-type (VT) serotypes. With a decrease in* S. pneumoniae* PCV7 VT serotypes, there was a corresponding increase in* H. influenzae,* particularly NTHi [100], exemplifying the dynamic, potentially competitive relationship between these two organisms [106–108]. The eradication of PCV7 VT in the nasopharynx has also been associated with higher rates of* H. influenzae* and* S. aureus* carriage in young children and infants, highlighting those virulent serotypes of* S. pneumoniae* also having a competitive relationship with* S. aureus* as well as* H. influenzae* [75, 105]. Incidences of bacterial cocarriage are important to report to inform future vaccine developments. This is important as the effect of vaccines targeting nasopharyngeal pathogens may produce indirect effects, as the ultimate balance between cocolonising organisms is unknown. The future of vaccination is under scrutiny with each vaccination implemented against specific serotypes or serogroups, as vaccine-type replacement is detected in the years following vaccination. When it is so difficult to predict the effects after vaccination of the target species, it is even more difficult to account for the indirect effects on cocolonising species.

- IPD remains an important disease both in the UK and worldwide responsible for morbidity and mortality.
- PCVs are currently the most effective pneumococcal vaccinations available at both reducing colonization of invasive serotypes and invasive disease.
- The role of conjugate vaccines in the nasopharyngeal niche remains unclear as studies initially focused on serotype/serogroup replacement of the target species.
- Serotype replacement occurs after PCV implementation, driving the need to develop new vaccination strategies independent of pneumococcal serotype inclusion.
- Bacterial carriage studies in the conjugate vaccine era have primarily focused on carriage of the target species; there have not been as many studies looking at nasopharyngeal cocarriage.
- Studies that have looked at nasopharyngeal cocarriage in the PCV era have shown changes in carriage of* H. influenzae* and* S. aureus* following PCV implementation, implying that a reduction in vaccine-type pneumococci will result in an increase of carried* H. influenzae* and* S. aureus*.
- It is currently difficult to define all potential indirect effects of PCV on the carriage of nonpneumococcal organisms.

## Figures and Tables

**Table 1:** Examples of studies identifying respiratory cocarriage of bacterial species in the PCV era.

Study	Study detail: vaccination status, species selected, and origin of samples	Methodology
Wiertsema et al. [104]	Post-PCV7, Spn, and Hflu (Australia)	Nasopharyngeal swab (NP) and conventional culture
Xu et al. [101]	Post-PCV7, Spn, Hflu, and Mcat (USA)	Nasal swab (NS), oropharyngeal sample (OP), and conventional culture
Spijkerman et al. [105]	Post-PCV7, Spn, SA, Hflu, and Mcat (Netherlands)	NP swab and conventional culture
Principi et al. [24]	Pre-PCV7, Spn, Hflu, and Mcat (Italy)	NP swab and conventional culture
Biesbroek et al. [75]	Post-PCV7 and pre-PCV7, Spn, Mcat, SA, and Spn (Netherlands)	NP swab and 454 pyrosequencing
Xu et al. [76]	Post-PCV7, Spn, Hflu, Mcat, and SA (USA)	NP, OP, and conventional culture
Pettigrew et al. [77]	Post-PCV7, Spn, Hflu, and Mcat (USA)	NP and 454 pyrosequencing
Laufer et al. [102]	Post-PCV7, Spn (USA)	NS swab and 454 pyrosequencing
Bogaert et al. [103]	Post-PCV7 and reduced dose PCV7, Spn, Hflu, Mcat, and SA (Netherlands)	NP swab and 454 pyrosequencing

This table includes vaccination status, species chosen for monitoring, and the origin of the samples as well as the methodology used for a comparison. Spn: *S. pneumoniae*; Hflu: *H. influenzae*; SA: *S. aureus*; Mcat: *M. catarrhalis*. 454 pyrosequencing is nonculture based identification.

## References

[1] Health Protection Agency https://www.gov.uk/government/collections/pneumococcal-disease-guidance-data-and-analysis.

[2] Centers for Disease Control and Prevention (2010). *ABCs Report: Streptococcus pneumoniae, 2010*.

[3] International Vaccine Access Centre (2014). *VIMS Report: Global Vaccine Introduction*.

[4] Tocheva A. S., Jefferies J. M. C., Rubery H. (2011). Declining serotype coverage of new pneumococcal conjugate vaccines relating to the carriage of Streptococcus pneumoniae in young children. *Vaccine*.

[5] Huang S. S., Platt R., Rifas-Shiman S. L., Pelton S. I., Goldmann D., Finkelstein J. A. (2001). Post-PCV7 changes in colonizing pneumococcal serotypes in 16 Massachusetts communities, 2001 and 2004. *Pediatrics*.

[6] Ansaldi F., de Florentiis D., Canepa P. (2012). Carriage of *Streptoccoccus pneumoniae* 7 years after implementation of vaccination program in a population with very high and long-lasting coverage, Italy. *Vaccine*.

[7] Miller E., Andrews N. J., Waight P. A., Slack M. P. E., George R. C. (2011). Herd immunity and serotype replacement 4 years after seven-valent pneumococcal conjugate vaccination in England and Wales: an observational cohort study. *The Lancet Infectious Diseases*.

[8] Keck J. W., Wenger J. D., Bruden D. L. (2014). PCV7-induced changes in pneumococcal carriage and invasive disease burden in Alaskan children. *Vaccine*.

[9] Choe Y. J., Choi E. H., Lee H. J. (2013). The changing epidemiology of childhood pneumococcal disease in Korea. *Infection and Chemotherapy*.

[10] Poehling K. A., Talbot T. R., Griffin M. R. (2006). Invasive pneumococcal disease among infants before and after introduction of pneumococcal conjugate vaccine. *The Journal of the American Medical Association*.

[11] van Hoek A. J., Sheppard C. L., Andrews N. J. (2014). Pneumococcal carriage in children and adults two years after introduction of the thirteen valent pneumococcal conjugate vaccine in England. *Vaccine*.

[12] Dagan R., Juergens C., Trammel J. (2015). Efficacy of 13-valent pneumococcal conjugate vaccine (PCV13) versus that of 7-valent PCV (PCV7) against nasopharyngeal colonization of antibiotic-nonsusceptible *Streptococcus pneumoniae*. *The Journal of Infectious Diseases*.

[13] Zuccotti G., Mameli C., Daprai L. (2014). Serotype distribution and antimicrobial susceptibilities of nasopharyngeal isolates of *Streptococcus pneumoniae* from healthy children in the 13-valent pneumococcal conjugate vaccine era. *Vaccine*.

[14] Choi Y. H., Jit M., Flasche S., Gay N., Miller E. (2012). Mathematical modelling long-term effects of replacing prevnar7 with prevnar13 on invasive pneumococcal diseases in england and wales. *PLoS ONE*.

[15] Turnbaugh P. J., Ley R. E., Hamady M., Fraser-Liggett C. M., Knight R., Gordon J. I. (2007). The human microbiome project. *Nature*.

[16] Peterson J., Garges S., Giovanni M. (2009). The NIH human microbiome project. *Genome Research*.

[17] Rodgers G. L., Klugman K. P. (2011). The future of pneumococcal disease prevention. *Vaccine*.

[18] Bratcher P. E., Kim K.-H., Kang J. H., Hong J. Y., Nahm M. H. (2010). Identification of natural pneumococcal isolates expressing serotype 6D by genetic, biochemical and serological characterization. *Microbiology*.

[19] Calix J. J., Nahm M. H. (2010). A new pneumococcal serotype, 11E, has a variably inactivated wcjE Gene. *Journal of Infectious Diseases*.

[20] Henrichsen J. (1995). Six newly recognized types of *Streptococcus pneumoniae*. *Journal of Clinical Microbiology*.

[21] In H. P., Pritchard D. G., Cartee R., Brandao A., Brandileone M. C. C., Nahm M. H. (2007). Discovery of a new capsular serotype (6C) within serogroup 6 of *Streptococcus pneumoniae*. *Journal of Clinical Microbiology*.

[22] Calix J. J., Porambo R. J., Brady A. M. (2012). Biochemical, genetic, and serological characterization of two capsule subtypes among *Streptococcus pneumoniae* serotype 20 strains: discovery of a new pneumococcal serotype. *The Journal of Biological Chemistry*.

[23] Aniansson G., Alm B., Andersson B. (1992). Nasopharyngeal colonization during the first year of life. *The Journal of Infectious Diseases*.

[24] Principi N., Marchisio P., Schito G. C., Mannelli S. (1999). Risk factors for carriage of respiratory pathogens in the nasopharynx of healthy children. *Pediatric Infectious Disease Journal*.

[25] Brueggemann A. B., Griffiths D. T., Meats E., Peto T., Crook D. W., Spratt B. G. (2003). Clonal relationships between invasive and carriage *Streptococcus pneumoniae* and serotype- and clone-specific differences in invasive disease potential. *Journal of Infectious Diseases*.

[26] Lipsitch M., Abdullahi O., D'Amour A. (2012). Estimating rates of carriage acquisition and clearance and competitive ability for pneumococcal serotypes in kenya with a markov transition model. *Epidemiology*.

[27] Douglas R. M., Hansman D., Miles H. B., Paton J. C. (1986). Pneumococcal carriage and type-specific antibody. Failure of a 14-valent vaccine to reduce carriage in healthy children. *The American Journal of Diseases of Children*.

[28] National Health Service (2010). *The Safest Way to Protect Our Children from Pneumococcal Disease*.

[29] Jackson L. A., Gurtman A., van Cleeff M. (2013). Immunogenicity and safety of a 13-valent pneumococcal conjugate vaccine compared to a 23-valent pneumococcal polysaccharide vaccine in pneumococcal vaccine-naive adults. *Vaccine*.

[30] Palmu A. A., Jokinen J., Borys D. (2013). Effectiveness of the ten-valent pneumococcal *Haemophilus influenzae* protein D conjugate vaccine (PHiD-CV10) against invasive pneumococcal disease: a cluster randomised trial. *The Lancet*.

[31] Reijtman V., Fossati S., Hernández C. (2013). Serotype distribution of pneumococci isolated from pediatric patients with acute otitis media and invasive infections, and potential coverage of pneumococcal conjugated vaccines. *Revista Argentina de Microbiologia*.

[32] Johnson H. L., Deloria-Knoll M., Levine O. S. (2010). Systematic evaluation of serotypes causing invasive pneumococcal disease among children under five: the pneumococcal global serotype project. *PLoS Medicine*.

[33] Centres for Disease Control and Prevention (2010). Prevention of pneumococcal disease among infants and children—use of 13-valent pneumococcal conjugate vaccine and 23-valent pneumococcal polysaccharide vaccine—recommendations of the Advisory Committee on Immunization Practices (ACIP). *Morbidity and Mortality Weekly Report*.

[34] Spratt B. G., Greenwood B. M. (2000). Prevention of pneumococcal disease by vaccination: does serotype replacement matter?. *The Lancet*.

[35] Jefferies J. M. C., Smith A., Clarke S. C., Dowson C., Mitchell T. J. (2004). Genetic analysis of diverse disease-causing pneumococci indicates high levels of diversity within serotypes and capsule switching. *Journal of Clinical Microbiology*.

[36] Wyres K. L., Lambertsen L. M., Croucher N. J. (2013). Pneumococcal capsular switching: a historical perspective. *The Journal of Infectious Diseases*.

[37] Porat N., Arguedas A., Spratt B. G. (2004). Emergence of penicillin-nonsusceptible *Streptococcus pneumoniae* clones expressing serotypes not present in the antipneumococcal conjugate vaccine. *The Journal of Infectious Diseases*.

[38] Temime L., Boelle P.-Y., Opatowski L., Guillemot D. (2008). Impact of capsular switch on invasive pneumococcal disease incidence in a vaccinated population. *PLoS ONE*.

[39] Muñoz-Almagro C., Jordan I., Gene A., Latorre C., Garcia-Garcia J. J., Pallares R. (2008). Emergence of invasive pneumococcal disease caused by nonvaccine serotypes in the era of 7-valent conjugate vaccine. *Clinical Infectious Diseases*.

[40] Kumar K. L., Ashok V., Ganaie F., Ramesh A. C. (2014). Nasopharyngeal carriage, antibiogram & serotype distribution of *Streptococcus pneumoniae* among healthy under five children. *Indian Journal of Medical Research*.

[41] Pimentel de Araujo F., D'Ambrosio F., Camilli R. (2014). Characterization of *Streptococcus pneumoniae* clones from paediatric patients with cystic fibrosis. *Journal of Medical Microbiology*.

[42] Lee G. M., Kleinman K., Pelton S. (2014). Impact of 13-valent pneumococcal conjugate vaccination on carriage in young children in Massachusetts. *Journal of the Pediatric Infectious Diseases Society*.

[43] Goldsmith C. E., Moore J. E., Murphy P. G. (1997). Pneumococcal resistance in the UK. *Journal of Antimicrobial Chemotherapy*.

[44] Torné A. N., Dias J. G., Quinten C. (2014). European enhanced surveillance of invasive pneumococcal disease in 2010: data from 26 European countries in the post-heptavalent conjugate vaccine era. *Vaccine*.

[45] Cho E. Y., Lee H., Choi E. H. (2014). Serotype distribution and antibiotic resistance of *Streptococcus pneumoniae* isolated from invasive infections after optional use of the 7-valent conjugate vaccine in Korea, 2006–2010. *Diagnostic Microbiology and Infectious Disease*.

[46] Keenan J. D., Klugman K. P., McGee L. (2015). Evidence for clonal expansion after antibiotic selection pressure: pneumococcal multilocus sequence types before and after mass azithromycin treatments. *Journal of Infectious Diseases*.

[47] Song J.-H., Dagan R., Klugman K. P., Fritzell B. (2012). The relationship between pneumococcal serotypes and antibiotic resistance. *Vaccine*.

[48] Leiberman A., Dagan R., Leibovitz E., Yagupsky P., Fliss D. (1999). The bacteriology of the nasopharynx in childhood. *International Journal of Pediatric Otorhinolaryngology*.

[49] Murphy T. F., Brauer A. L., Grant B. J. B., Sethi S. (2005). *Moraxella catarrhalis* in chronic obstructive pulmonary disease: burden of disease and immune response. *The American Journal of Respiratory and Critical Care Medicine*.

[50] Parameswaran G. I., Wrona C. T., Murphy T. F., Sethi S. (2009). *Moraxella catarrhalis* acquisition, airway inflammation and protease-antiprotease balance in chronic obstructive pulmonary disease. *BMC Infectious Diseases*.

[51] Jerng J.-S., Hsueh P.-R., Teng L.-J., Lee L.-N., Yang P.-C., Luh K.-T. (1997). Empyema thoracis and lung abscess caused by viridans streptococci. *American Journal of Respiratory and Critical Care Medicine*.

[52] Brown A. F., Leech J. M., Rogers T. R., McLoughlin R. M. (2014). *Staphylococcus aureus* colonization: modulation of host immune response and impact on human vaccine design. *Frontiers in Immunology*.

[53] Bogaert D., Hermans P. W. M., Boelens H. (2005). Epidemiology of nasopharyngeal carriage of *Neisseria meningitidis* in healthy Dutch children. *Clinical Infectious Diseases*.

[54] Darton T., Guiver M., Naylor S. (2009). Severity of meningococcal disease associated with genomic bacterial load. *Clinical Infectious Diseases*.

[55] Meats E., Feil E. J., Stringer S. (2003). Characterization of encapsulated and noncapsulated *Haemophilus influenzae* and determination of phylogenetic relationships by multilocus sequence typing. *Journal of Clinical Microbiology*.

[56] Zhang L., Xie J., Patel M. (2012). Nontypeable *Haemophilus influenzae* genetic islands associated with chronic pulmonary infection. *PLoS ONE*.

[57] van Wessel K., Rodenburg G. D., Veenhoven R. H., Spanjaard L., van der Ende A., Sanders E. A. M. (2011). Nontypeable haemophilus influenzae invasive disease in The Netherlands: a retrospective surveillance study 2001–2008. *Clinical Infectious Diseases*.

[58] Ghaffar F., Friedland I. R., Mccracken G. H. (1999). Dynamics of nasopharyngeal colonization by *Streptococcus pneumoniae*. *Pediatric Infectious Disease Journal*.

[59] Tocheva A. S., Jefferies J. M. C., Christodoulides M., Faust S. N., Clarke S. C. (2013). Distribution of carried pneumococcal clones in UK children following the introduction of the 7-valent pneumococcal conjugate vaccine: a 3-year cross-sectional population based analysis. *Vaccine*.

[60] Hussain M., Melegaro A., Pebody R. G. (2005). A longitudinal household study of *Streptococcus pneumoniae* nasopharyngeal carriage in a UK setting. *Epidemiology and Infection*.

[61] Ala'aldeen D. A. A., Neal K. R., Ait-Tahar K. (2000). Dynamics of meningococcal long-term carriage among university students and their implications for mass vaccination. *Journal of Clinical Microbiology*.

[62] Millar M. R., Walsh T. R., Linton C. J., Zhang S., Leemingn J. P., Bennett P. M. (2001). Carriage of antibiotic-resistant bacteria by healthy children. *Journal of Antimicrobial Chemotherapy*.

[63] Mobbs K. J., van Saene H. K. F., Sunderland D., Davies P. D. O. (1999). Oropharyngeal Gram-negative bacillary carriage in chronic obstructive pulmonary disease: relation to severity of disease. *Respiratory Medicine*.

[64] Andrews N., Waight P. A., Borrow R. (2011). Using the indirect cohort design to estimate the effectiveness of the seven valent pneumococcal conjugate vaccine in England and Wales. *PLoS ONE*.

[65] Hanage W. P., Finkelstein J. A., Huang S. S. (2010). Evidence that pneumococcal serotype replacement in Massachusetts following conjugate vaccination is now complete. *Epidemics*.

[66] Cauchemez S., Temime L., Valleron A.-J. (2006). *S. pneumoniae* transmission according to inclusion in conjugate vaccines: Bayesian analysis of a longitudinal follow-up in schools. *BMC Infectious Diseases*.

[67] Tocheva A. S., Jefferies J. M. C., Christodoulides M., Faust S. N., Clarke S. C. (2010). Increase in serotype 6C pneumococcal carriage, United Kingdom. *Emerging Infectious Diseases*.

[68] Bijlsma M. W., Brouwer M. C., Spanjaard L., van de Beek D., van der Ende A. (2014). A decade of herd protection after introduction of meningococcal serogroup C conjugate vaccination. *Clinical Infectious Diseases*.

[69] Coughtrie A. L., Whittaker R. N., Begum N. (2014). Evaluation of swabbing methods for estimating the prevalence of bacterial carriage in the upper respiratory tract: a cross sectional study. *BMJ Open*.

[70] Warnke P., Frickmann H., Ottl P., Podbielski A., Rohde H. (2014). Nasal screening for MRSA: different swabs—different results!. *PLoS ONE*.

[71] Gladstone R. A., Jefferies J. M., Faust S. N., Clarke S. C. (2012). Sampling methods for the study of pneumococcal carriage: a systematic review. *Vaccine*.

[72] Satzke C., Turner P., Virolainen-Julkunen A. (2013). Standard method for detecting upper respiratory carriage of Streptococcus pneumoniae: updated recommendations from the World Health Organization Pneumococcal Carriage Working Group. *Vaccine*.

[73] Flasche S., van Hoek A. J., Sheasby E. (2011). Effect of pneumococcal conjugate vaccination on serotype-specific carriage and invasive disease in England: a cross-sectional study. *PLoS Medicine*.

[74] Klugman K. P. (2001). Efficacy of pneumococcal conjugate vaccines and their effect on carriage and antimicrobial resistance. *The Lancet Infectious Diseases*.

[75] Biesbroek G., Wang X., Keijser B. J. F. (2014). Seven-valent pneumococcal conjugate vaccine and nasopharyngeal microbiota in healthy children. *Emerging Infectious Diseases*.

[76] Xu Q., Almudervar A., Casey J. R., Pichichero M. E. (2012). Nasopharyngeal bacterial interactions in children. *Emerging Infectious Diseases*.

[77] Pettigrew M. M., Laufer A. S., Gent J. F., Kong Y., Fennie K. P., Metlay J. P. (2012). Upper respiratory tract microbial communities, acute otitis media pathogens, and antibiotic use in healthy and sick children. *Applied and Environmental Microbiology*.

[78] Rodrigues F., Morales-Aza B., Turner K. M. E. (2013). Multiple *Streptococcus pneumoniae* serotypes in aural discharge samples from children with acute otitis media with spontaneous otorrhea. *Journal of Clinical Microbiology*.

[79] Ruohola A., Meurman O., Nikkari S. (2006). Microbiology of acute otitis media in children with tympanostomy tubes: prevalences of bacteria and viruses. *Clinical Infectious Diseases*.

[80] García-Rodríguez J. Á., Fresnadillo Martínez M. J. (2002). Dynamics of nasopharyngeal colonization by potential respiratory pathogens. *Journal of Antimicrobial Chemotherapy*.

[81] Lysenko E. S., Clarke T. B., Shchepetov M. (2007). Nod1 signaling overcomes resistance of *S. pneumoniae* to opsonophagocytic killing. *PLoS Pathogens*.

[82] Lysenko E. S., Lijek R. S., Brown S. P., Weiser J. N. (2010). Within-host competition drives selection for the capsule virulence determinant of *Streptococcus pneumoniae*. *Current Biology*.

[83] Jartti T., Kuneinen S., Lehtinen P. (2011). Nasopharyngeal bacterial colonization during the first wheezing episode is associated with longer duration of hospitalization and higher risk of relapse in young children. *European Journal of Clinical Microbiology & Infectious Diseases*.

[84] http://www.meningitis.org/disease-info/vaccines.

[85] Turk D. C. (1984). The pathogenicity of *Haemophilus influenzae*. *Journal of Medical Microbiology*.

[86] Heath P. T., McVernon J. (2002). The UK hib vaccine experience. *Archives of Disease in Childhood*.

[87] Galil K., Singleton R., Levine O. S. (1999). Reemergence of invasive *Haemophilus influenzae* type b disease in a well-vaccinated population in remote Alaska. *Journal of Infectious Diseases*.

[88] Ladhani S. N., Collins S., Vickers A. (2012). Invasive *Haemophilus influenzae* serotype e and f disease, England and Wales. *Emerging Infectious Diseases*.

[89] Ribeiro G. S., Reis J. N., Cordeiro S. M. (2003). Prevention of *Haemophilus influenzae* type b (Hib) meningitis and emergence of serotype replacement with type a strains after introduction of Hib immunization in Brazil. *The Journal of Infectious Diseases*.

[90] Adam H. J., Richardson S. E., Jamieson F. B., Rawte P., Low D. E., Fisman D. N. (2010). Changing epidemiology of invasive *Haemophilus influenzae* in Ontario, Canada: evidence for herd effects and strain replacement due to Hib vaccination. *Vaccine*.

[91] Shuel M., Hoang L., Law D. K. S., Tsang R. (2011). Invasive *Haemophilus influenzae* in British Columbia: non-Hib and non-typeable strains causing disease in children and adults. *International Journal of Infectious Diseases*.

[92] Brogden K. A., Guthmiller J. M., Taylor C. E. (2005). Human polymicrobial infections. *The Lancet*.

[93] Peleg A. Y., Hogan D. A., Mylonakis E. (2010). Medically important bacterialg-fungal interactions. *Nature Reviews Microbiology*.

[94] Heikkinen T., Chonmaitree T. (2003). Importance of respiratory viruses in acute otitis media. *Clinical Microbiology Reviews*.

[95] Costerton J. W., Cheng K. J., Geesey G. G. (1987). Bacterial biofilms in nature and disease. *Annual Review of Microbiology*.

[96] Hall-Stoodley L., Costerton J. W., Stoodley P. (2004). Bacterial biofilms: from the natural environment to infectious diseases. *Nature Reviews Microbiology*.

[97] Hoa M., Tomovic S., Nistico L. (2009). Identification of adenoid biofilms with middle ear pathogens in otitis-prone children utilizing SEM and FISH. *International Journal of Pediatric Otorhinolaryngology*.

[98] Hall-Stoodley L., Hu F. Z., Gieseke A. (2006). Direct detection of bacterial biofilms on the middle-ear mucosa of children with chronic otitis media. *Journal of the American Medical Association*.

[99] Saafan M. E., Ibrahim W. S., Tomoum M. O. (2013). Role of adenoid biofilm in chronic otitis media with effusion in children. *European Archives of Oto-Rhino-Laryngology*.

[100] Wiertsema S. P., Kirkham L.-A. S., Corscadden K. J. (2011). Predominance of nontypeable *Haemophilus influenzae* in children with otitis media following introduction of a 3 + 0 pneumococcal conjugate vaccine schedule. *Vaccine*.

[101] Xu Q., Casey J. R., Chang A., Pichichero M. E. (2012). When co-colonizing the nasopharynx *Haemophilus influenzae* predominates over *Streptococcus pneumoniae* except serotype 19A strains to cause acute otitis media. *Pediatric Infectious Disease Journal*.

[102] Laufer A. S., Metlay J. P., Gent J. F., Fennie K. P., Kong Y., Pettigrewa M. M. (2011). Microbial communities of the upper respiratory tract and otitis media in children. *mBio*.

[103] Bogaert D., Keijser B., Huse S. (2011). Variability and diversity of nasopharyngeal microbiota in children: a metagenomic analysis. *PLoS ONE*.

[104] Wiertsema S. P., Kirkham L.-A. S., Corscadden K. J. (2011). Predominance of nontypeable *Haemophilus influenzae* in children with otitis media following introduction of a 3 + 0 pneumococcal conjugate vaccine schedule. *Vaccine*.

[105] Spijkerman J., Prevaes S. M. P. J., van Gils E. J. M. (2012). Long-term effects of pneumococcal conjugate vaccine on nasopharyngeal carriage of *S. pneumoniae*, *S. aureus*, *H. influenzae* and *M. catarrhalis*. *PLoS ONE*.

[106] Lysenko E. S., Ratner A. J., Nelson A. L., Weiser J. N. (2005). The role of innate immune responses in the outcome of interspecies competition for colonization of mucosal surfaces. *PLoS Pathogens*.

[107] Shakhnovich E. A., King S. J., Weiser J. N. (2002). Neuraminidase expressed by *Streptococcus pneumoniae* desialylates the lipopolysaccharide of *Neisseria meningitidis* and *Haemophilus influenzae*: a paradigm for interbacterial competition among pathogens of the human respiratory tract. *Infection and Immunity*.

[108] Ratner A. J., Lysenko E. S., Paul M. N., Weiser J. N. (2005). Synergistic proinflammatory responses induced by polymicrobial colonization of epithelial surfaces. *Proceedings of the National Academy of Sciences of the United States of America*.

